# Therapeutic Value of Nitrate of Lead

**Published:** 1878-10

**Authors:** 


Therapeatic Value of Nitrate of Lead.—The late Dr. Madison Marsh, of Louisiana, a few years ago urged upon the profession, through the columns of this journal, the very great value of the nitrate of lead in many skin affections and superficial erosions. Some recent observations of an Italian physician, Dr. Galletti, add further evidence on this point. This writer states that he has recently effected a cure in three cases of epithelioma, in one of which the part affected was the nose, in a second the cheek, and the third the sternum. The mode in which he applied the remedy was by dusting the powder over the affected part, and recovery took place when this had been done about four times. Two obstinate ulcers of the foot, which had proved rebellious to other methods, quickly recovered under the same treatment. Dr. Vanzetti has recently recommended the use of the nitrate of lead in onychia maligna.—*Med. & Surg. Rep.*, August 10, 1878.

